# Micromechanical Modeling of the Biaxial Deformation-Induced Phase Transformation in Polyethylene Terephthalate

**DOI:** 10.3390/polym14153028

**Published:** 2022-07-26

**Authors:** Fateh Enouar Mamache, Amar Mesbah, Hanbing Bian, Fahmi Zaïri

**Affiliations:** 1 Laboratory of Advanced Mechanics, University of Sciences and Technology Houari Boumediene, Algiers 16111, Algeria; mamacheanouar1991@gmail.com (F.E.M.); ammar_mesbah@yahoo.fr (A.M.); 2 Laboratoire de Génie Civil et géo-Environnement, Université de Lille, IMT Nord Europe, JUNIA, Université d’Artois, ULR 4515-LGCgE, F-59000 Lille, France; hanbing.bian@univ-lille.fr

**Keywords:** crystallizable PET, micromechanical model, viscoplasticity, temperature effect, biaxial loading

## Abstract

In this paper, a micromechanics-based constitutive representation of the deformation-induced phase transformation in polyethylene terephthalate is proposed and verified under biaxial loading paths. The model, formulated within the Eshelby inclusion theory and the micromechanics framework, considers the material system as a two-phase medium, in which the active interactions between the continuous amorphous phase and the discrete newly formed crystalline domains are explicitly considered. The Duvaut–Lions viscoplastic approach is employed in order to introduce the rate-dependency of the yielding behavior. The model parameters are identified from uniaxial data in terms of stress–strain curves and crystallization kinetics at two different strain rates and two different temperatures above glass transition temperature. Then, it is shown that the model predictions are in good agreement with available experimental results under equal biaxial and constant width conditions. The role of the crystallization on the intrinsic properties is emphasized thanks to the model considering the different loading parameters in terms of mechanical path, strain rate and temperature.

## 1. Introduction

Polyethylene terephthalate (PET) is one of the most used engineering polymers in applications where lightweight, chemical resistance, mechanical strength and thermal resistance are required. PET is a well-known example of a material system exhibiting a crystallization induced by mechanical loading. The phase transformation, highly dependent on the loading path, loading rate and temperature, implies a modification of the material response at the macroscale. Establishment of the structure–property relationship is a prerequisite for a reliable assessment of the material design whether under in-service or the manufacturing (e.g., hot-drawing near glass transition) process. Over the years, rheologically based models were formulated to reproduce the mechanical response of PET [1,2,3,4,5,6,7,8,9,10,11,12] and other crystallizable thermoplastics [13,14]. From the rheological viewpoint, these models combine different resistances to represent intermolecular and molecular network micromechanisms. The strain stiffening due to the appearance of the newly formed crystalline phase is introduced either implicitly via threshold conditions or explicitly by considering the crystallization kinetics via an Avrami expression. In the latter approach, the presence of crystallites is introduced thanks to the concept of volume fraction in which the polymer is seen as a two-phase composite. The concept was also extended to semicrystalline polymers [15,16,17,18,19]. Nonetheless, the active interactions between the continuous amorphous phase and the discrete crystalline domains are not considered, which constitutes a weakness from the physical viewpoint. Only micromechanical homogenization methods allow to incorporate local interactions and then lead to a physically convenient way to constitutively represent the heterogeneous problem. The latter is the basis of two main approaches to predict the effective mechanical properties of semicrystalline media, with different idealizations of the microstructure to derive the constitutive relations. In a first approach, the material can be defined as an aggregate of layered two-phase composite inclusions, each one represented by an amorphous layer and a crystalline lamella. The approach has been used in a viscoplastic framework [20,21], an elasto-viscoplastic framework [22,23,24,25] or a purely elastic framework [26,27]. In a second approach, the material system is treated as an Eshelby inclusion problem by seeing the crystals as reinforcing ellipsoidal inclusions embedded into a continuous amorphous matrix. The Eshelby-type inclusion approach was mainly used for the initial elastic behavior [26,28,29,30,31,32,33], the linear viscoelastic behavior [34], the initial yield behavior [35] and the postyield behavior [36].

The main objective of this paper is to explore the relevance of a micromechanics-based elasto-viscoplastic modeling to represent the deformation-induced phase transformation in PET. The model arises from the Eshelby inclusion theory and the micromechanics framework. The intrinsic viscosity of the amorphous phase is considered using the Duvaut–Lions viscoplastic approach, and the crystallization kinetics is governed by an Avrami equation. The model parameters are calibrated using available uniaxial data of a PET stretched under two different strain rates at two different temperatures above glass transition temperature. The model capacities to predict the mechanical response along with the phase transformation are verified by comparison with the available experimental data of a PET stretched under equal biaxial and constant width conditions.

The outline of the present paper is as follows. The micromechanical model is presented in Section 2. Section 3 is devoted to the comparison of the model simulations with experimental observations. Concluding remarks are given in Section 4.

## 2. Model

The constitutive representation of the PET system is treated as an Eshelby-type inclusion problem in which the material volume element consists of an amorphous phase as the continuous phase and discrete newly born crystalline domains. The two constitutive phases are supposed to be isotropic and homogeneous media with elastic stiffness tensors $C_{am}$ and $C_{cry}$.

### 2.1. Micromechanics-Based Theory for Deformation-Induced Phase Transformation

The constitutive relation between the macrostress tensor $\bar{\sigma }$ and the elastic part ${\bar{\varepsilon }}^{e}$ of the macrostrain tensor $\bar{\varepsilon }$ is given by:

(1) $$\bar{\sigma }=\bar{C}:{\bar{\varepsilon }}^{e}$$

in which $\bar{C}$ is the macroscopic elastic stiffness tensor of the semicrystalline material expressed as [37]:

(2) $$\bar{C}=C_{am}.\left[I-Y.{\left(S.Y+I\right)}^{-1}\right]$$

where I is the identity tensor, S is the Eshelby tensor and Y is a tensor expressed as:

(3) $$Y=-\phi_{cry}{\left[S+{\left(C_{cry}-C_{am}\right)}^{-1}.C_{am}\right]}^{-1}$$

where $\phi_{cry}$ is the volume fraction of the strain-induced crystalline phase for which the kinetics is governed by an Avrami-type formula that is specified below.

The fourth-order isotropic elastic stiffness tensors, $C_{am}$ and $C_{cry}$, are expressed, in Cartesian components, as follows:

(4) $${\left(C_{am}\right)}_{ijkl}=\frac{E_{am}}{2\left(1+\nu_{am}\right)}\left[\left(\delta_{ik}\delta_{jl}+\delta_{il}\delta_{jk}\right)+\frac{2\nu_{am}}{1-2\nu_{am}}\delta_{ij}\delta_{kl}\right]$$

(5) $${\left(C_{cry}\right)}_{ijkl}=\frac{E_{cry}}{2\left(1+\nu_{cry}\right)}\left[\left(\delta_{ik}\delta_{jl}+\delta_{il}\delta_{jk}\right)+\frac{2\nu_{cry}}{1-2\nu_{cry}}\delta_{ij}\delta_{kl}\right]$$

in which $E_{am}$ and $E_{cry}$ are the Young’s moduli, and $\nu_{am}$ and $\nu_{cry}$ are the Poisson’s ratios. The term $\delta_{ij}$ denotes the Kronecker delta symbol.

The plastic yielding is considered from the continuum plasticity theory. Regarding the associative plastic flow rule, the macroscopic plastic strain rate ${\dot{\bar{\varepsilon }}}^{p}$ is expressed as:

(6) $${\dot{\bar{\varepsilon }}}^{p}=\dot{\lambda }\frac{\partial \bar{F}}{\partial \bar{\sigma }}=\left(1-\phi_{cry}\right)\dot{\lambda }\frac{\bar{T}:\bar{\sigma }}{\sqrt{\bar{\sigma }:\bar{T}:\bar{\sigma }}}$$

where $\dot{\lambda }$ is the plastic multiplier and $\bar{F}$ is the macroscopic yield function. Considering the von Mises yield criterion with isotropic plastic hardening for the continuous amorphous phase, the yield function $\bar{F}$ can be expressed as [38]:

(7) $$\bar{F}=(1-\phi_{cry})\sqrt{\bar{\sigma }:\bar{T}:\bar{\sigma }}-\sqrt{\frac{2}{3}}[\sigma_{y}+h{\left({\bar{e}}^{p}\right)}^{q}]\leq 0$$

in which ${\bar{e}}^{p}$ is the macroscopic equivalent plastic strain, $\sigma_{y}$ is the initial yield strength of the amorphous phase and, h and q are the hardening parameters of the amorphous phase. The term $\bar{T}$ can be given by:

(8) $${\bar{T}}_{ijkl}={\bar{T}}_{1}\delta_{ij}\delta_{kl}+{\bar{T}}_{2}\left(\delta_{ik}\delta_{jl}+\delta_{il}\delta_{jk}\right)$$

where ${\bar{T}}_{1}$ and ${\bar{T}}_{2}$ are provided in Appendix A.

The volume fraction of the strain-induced crystalline phase $\phi_{cry}$ is given by:

(9) $$\phi_{cry}=\phi_{\infty \_cry}\kappa$$

in which $\phi_{\infty \_cry}$ is the maximum crystal degree and κ is the total degree of transformation following the Avrami-type expression [4] modified by Ahzi et al. [5]:

(10) $$\dot{\kappa }=\frac{\dot{\varepsilon }}{{\dot{\varepsilon }}_{ref}}\alpha_{A}K_{av}{\left(-\ln\left(1-\kappa \right)\right)}^{\frac{\alpha_{A}-1}{\alpha_{A}}}\left(1-\kappa \right)$$

where $\alpha_{A}$ is the Avrami exponent, $K_{av}$ is the phase transformation rate function, $\dot{\varepsilon }$ is the applied strain rate and ${\dot{\varepsilon }}_{ref}$ is the reference strain rate.

The transformation rate function $K_{av}$ takes the empirical form defined as follows:

(11) $$K_{av}=1.47\times 10^{-3}{\left(\frac{4\pi Nu}{3\phi_{\infty \_cry}}\right)}^{1/3}\exp\left(-{\left(\frac{\theta -141}{47.33}\right)}^{2}\right)$$

in which Nu is the number density of nuclei in the amorphous phase.

This empirical formula first emerged for the study of spherulitic growth in thermally-induced crystallization and may not be an optimized choice for all kinetics of newly formed crystals due to differences in morphology and in size. Nonetheless, it was also employed in previous studies in the context of strain-induced crystallization in PET [5,6] and in PLA [13,14] as a phenomenological description of the evolution of a newly formed phase.

### 2.2. Model Implementation

The model allows to relate elasto-viscoplastic macrobehavior to microstructure variations depending on the loading parameters in terms of mechanical path, strain rate and temperature. It is identified using uniaxial (UA) data and its predictability is verified using equal biaxial (EB) and constant width (CW) data.

The components of the macrostress tensor $\bar{\sigma }$ under a general biaxial stretching are:

(12) $${\bar{\sigma }}_{11}>0,\;{\bar{\sigma }}_{22}=R{\bar{\sigma }}_{11}\mathrm{and}\;{\bar{\sigma }}_{ij}=0\;\mathrm{for}\;\mathrm{all}\;\mathrm{other}\;\mathrm{components}$$

where $R={\bar{\sigma }}_{22}/{\bar{\sigma }}_{11}$ is the stress biaxial ratio:

(13) $$\begin{array}{c} \mathrm{UA}:\\ \mathrm{EB}:\\ \mathrm{CW}: \end{array}\begin{array}{c} R=0\\ R=1\\ 0<R<1 \end{array},$$

The CW condition is a particular biaxial loading in which a stretching is performed in one direction while the transversal one is kept constant. In this regard, the stress biaxial ratio is not constant but changes iteratively during the loading.

The terms $\bar{T}:\bar{\sigma }$ and $\bar{\sigma }:\bar{T}:\bar{\sigma }$ in Equation (6) write:

(14) $$\bar{T}:\bar{\sigma }={\bar{\sigma }}_{11}\mathrm{diag}\left({\bar{T}}_{1}+R{\bar{T}}_{1}+2{\bar{T}}_{2},{\bar{T}}_{1}+R{\bar{T}}_{1}+2R{\bar{T}}_{2}\right)$$

(15) $$\bar{\sigma }:\bar{T}:\bar{\sigma }={\bar{\sigma }}_{11}^{2}\left[{\bar{T}}_{1}{\left(1+R\right)}^{2}+2{\bar{T}}_{2}{\left(1+R\right)}^{2}\right]={\bar{\sigma }}_{11}^{2}\Phi \left(R\right)$$

The macroscopic plastic strain rate ${\dot{\bar{\varepsilon }}}^{p}$ becomes:

(16) $${\dot{\bar{\varepsilon }}}^{p}=\left(1-\phi_{cry}\right)\frac{\dot{\lambda }}{\sqrt{\Phi \left(R\right)}}\mathrm{diag}\left({\bar{T}}_{1}+R{\bar{T}}_{1}+2{\bar{T}}_{2},{\bar{T}}_{1}+R{\bar{T}}_{1}+2R{\bar{T}}_{2}\right)$$

**Figure 1:**
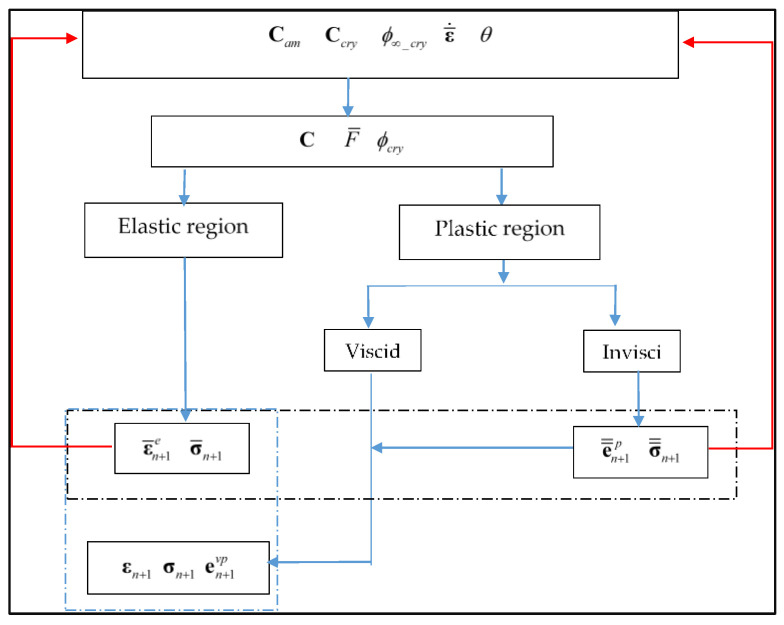
Algorithm of the model implementation.

The plastic multiplier $\dot{\lambda }$ was computed from the plastic consistency condition: $\dot{\lambda }\langle \dot{\bar{F}}\rangle =0$, the yield condition being formulated in a Kuhn–Tucker form by: $\dot{\lambda }\geq 0$, $\langle \bar{F}\rangle \leq 0$, $\dot{\lambda }\langle \bar{F}\rangle =0$. The model was coded in Matlab software using the flowchart provided in Figure 1.

The model inputs are the material constants, in particular the components of the amorphous elastic stiffness tensor, the components of the crystalline elastic stiffness tensor and the maximum crystal degree. The loading parameters, in terms of strain rate and temperature, are also specified. Both stiffness and yield function are updated iteratively while, once the threshold is reached, the crystallization amount increases with the mechanical loading. The rate-dependency of the yielding behavior is also taken into account by using the Duvaut–Lions viscoplastic approach [39,40]:

(17) $${\dot{\varepsilon }}^{vp}=\frac{1}{\eta }C_{am}^{-1}:(\bar{\sigma }-\overset{=}{\sigma })$$

(18) $${\dot{e}}^{vp}=\frac{1}{\eta }\left({\bar{e}}^{vp}-{\overset{=}{e}}^{p}\right)$$

where η is a viscosity parameter, $\bar{\sigma }$ and $\overset{=}{\sigma }$ are the total average viscoplastic stress tensor and the overall inviscid plastic stress tensor, respectively, and ${\bar{e}}^{vp}$ and ${\overset{=}{e}}^{p}$ are the viscoplastic strain tensor and the inviscid plastic strain tensor, respectively. The inviscid solution, in terms of the actual stress tensor ${\overset{=}{\sigma }}_{n+1}$ and the internal variable ${\overset{=}{e}}_{n+1}^{p}$, is updated at each increment allowing the calculation of the new stress ${\bar{\sigma }}_{n+1}$ and the viscoplastic strain ${\bar{e}}_{n+1}^{vp}$ by integrating the two previous equations using a backward Euler algorithm:

(19) $${\bar{\sigma }}_{n+1}=\frac{\left({\bar{\sigma }}_{n}+C_{am}:\Delta {\bar{\varepsilon }}_{n+1}\right)+\frac{\Delta t_{n+1}}{\eta }{\overset{=}{\sigma }}_{n+1}}{1+\frac{\Delta t_{n+1}}{\eta }}$$

(20) $${\bar{e}}_{n+1}^{vp}=\frac{{\bar{e}}_{n}^{vp}+\frac{\Delta t_{n+1}}{\eta }{\overset{=}{e}}_{n+1}^{p}}{1+\frac{\Delta t_{n+1}}{\eta }}$$

where $\Delta t_{n+1}$ is the time step. When $\Delta t_{n+1}/\eta \to \infty$, the inviscid solution is recovered, and when $\Delta t_{n+1}/\eta \to 0$, the elastic solution is achieved.

## 3. Results and Discussion

In what follows, the model is quantitatively compared with experimental results of PET loaded at different loading conditions. The model parameters for the two constitutive phases are listed in Table 1 and Table 2.

### 3.1. Model Identification

#### 3.1.1. Continuous Amorphous Phase Properties

**Figure 2:**
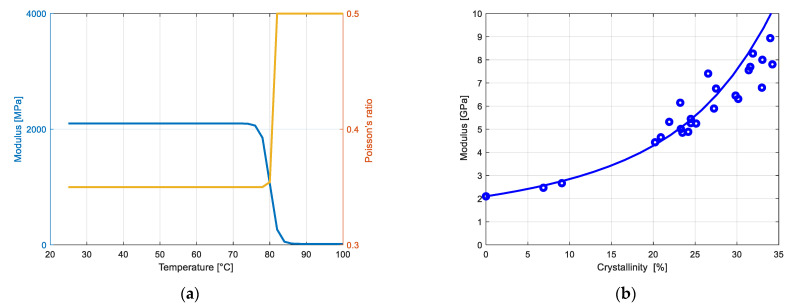
Model results of the linear elastic response: (**a**) amorphous elastic constants as a function of temperature, (**b**) overall modulus as a function of crystal content; solid lines: model; symbols: experimental data of Cosson et al. [11].

Figure 2a shows the variation with temperature θ of the amorphous stiffness $E_{am}$ around the glass transition temperature $\theta_{g}$. Like all amorphous polymers, the PET elastic modulus exhibits a plateau within the glassy state, a drastic drop within the glass transition region and a continued decrease within the rubbery region. This behavior is represented by the following function [8]:

(21) $$E_{am}\left(\theta \right)=\frac{1}{2}\left(E_{g}+E_{r}\right)-\frac{1}{2}\left(E_{g}-E_{r}\right)\tanh\left(\frac{5}{\Delta \theta }\left(\theta -\theta_{g}\right)\right)+X_{g}\left(\theta -\theta_{g}\right)$$

where $E_{g}$ is the amorphous modulus in the glassy region, $E_{r}$ is the amorphous modulus in the rubbery region, Δθ is the interval of the temperature range across which the glass transition occurs and $X_{g}$ is the slope outside the glass transition region. The variation with temperature θ of the amorphous Poisson’s ratio $\nu_{am}$ around $\theta_{g}$ is given by the following function:

(22) $$\nu_{am}\left(\theta \right)=\nu_{g}+\left(\nu_{r}-\nu_{g}\right)\exp\left(\frac{\theta -{\left(2\left(\theta_{g}+\Delta \theta -\theta \right)\right)}^{2}}{\theta_{g}+\Delta \theta }\right)\mathrm{for}\;\theta <\theta_{g},$$

(23) $$\nu_{am}\left(\theta \right)=\nu_{r}\;\mathrm{for}\;\theta \geq \theta_{g},$$

where $\nu_{g}$ is the amorphous Poisson’s ratio in the glassy region and $\nu_{r}$ is the amorphous Poisson’s ratio in the rubbery region.

#### 3.1.2. Discrete Crystalline Phase Properties

In the identification exercise, the crystal features, in terms of shape factor and elastic properties, are determined using the overall elastic properties at ambient temperature taken from the work of Cosson et al. [11] on previously crystallized PET. The theoretical and experimental stiffening is shown in Figure 2b as a function of crystal amount. Note that the amorphous elastic properties are considered to be independent of crystal amount.

#### 3.1.3. Overall Response

**Figure 3:**
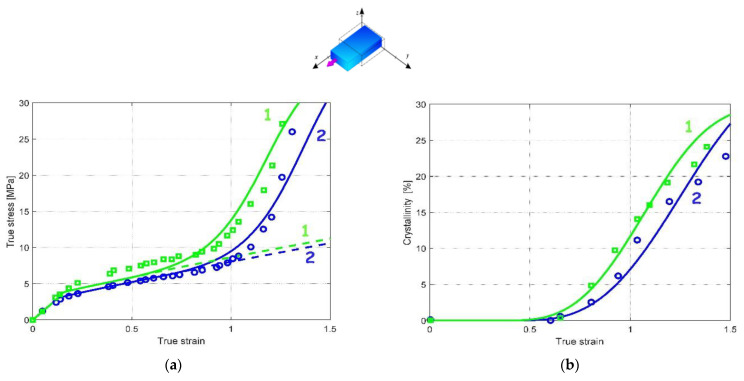
Model results under UA loading at a loading temperature of 90 °C and different strain rates (1: 2.1/s, 2: 0.42/s): (**a**) stress–strain response, (**b**) strain-induced crystallization; solid lines: model; dashed lines: model with no crystallization; symbols: experimental data of Salem [41].

The other model parameters were identified using the UA data of Salem [41] in terms of stress–strain and crystallization curves. The identification exercise includes the rate effect using two available strain rates: 0.42/s and 2.1/s. Only the straining temperature of 90 °C is used for the identification. Figure 3 presents the model results in comparison with the experimental stress–strain and crystallization curves. The solid lines represent the model results while the symbols designate the experimental data.

It can be observed that the model is able to reproduce adequately the strain rate dependency on the overall UA response along with the crystallization. The crystallization kinetics and the intrinsic viscosity of the amorphous matrix are the two rate-dependent factors introduced in the model and affecting the overall response. The increase in strain rate slightly affects the initial yield region but significantly influences the progressive strain hardening and the dramatic strain hardening occurring at a strain of approximately 1. Moreover, the model is able to capture the acceleration of both the crystallization onset and the crystallization amount with the strain rate.

### 3.2. Model Prediction

#### 3.2.1. Comparison between Model and Experiments

**Figure 4:**
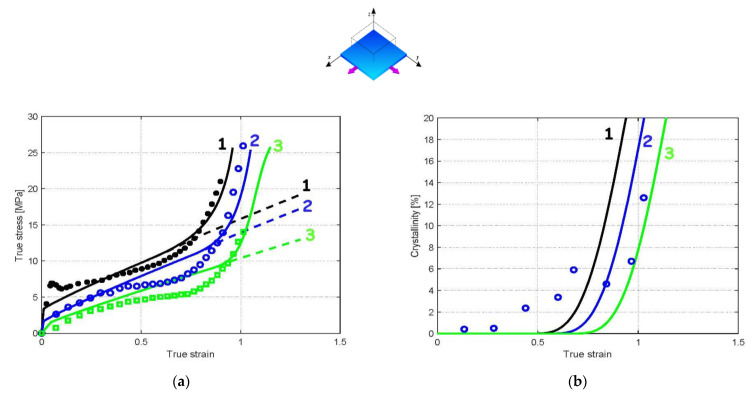
Model results under EB loading at a strain rate of 1/s and different loading temperatures (1: 83 °C, 2: 89 °C, 3: 94 °C): (**a**) stress–strain response, (**b**) strain-induced crystallization; solid lines: model; dashed lines: model with no crystallization; symbols: experimental data of Adams et al. [2].

**Figure 5:**
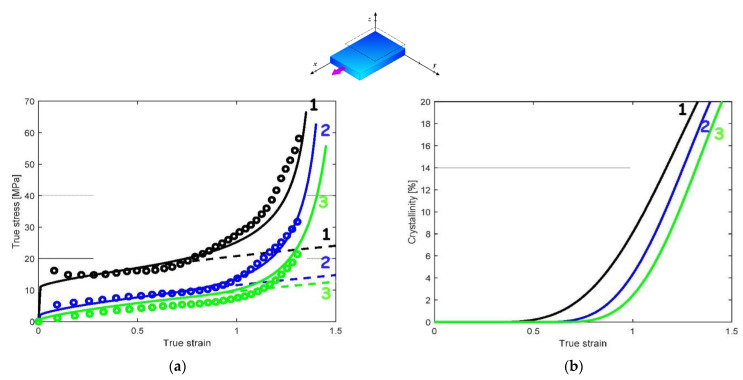
Model results under CW loading at a strain rate of 1 /s and different loading temperatures (1: 80 °C, 2: 86 °C, 3: 92 °C): (**a**) stress–strain response, (**b**) strain-induced crystallization; solid lines: model; dashed lines: model with no crystallization; symbols: experimental data of Adams et al. [2].

**Figure 6:**
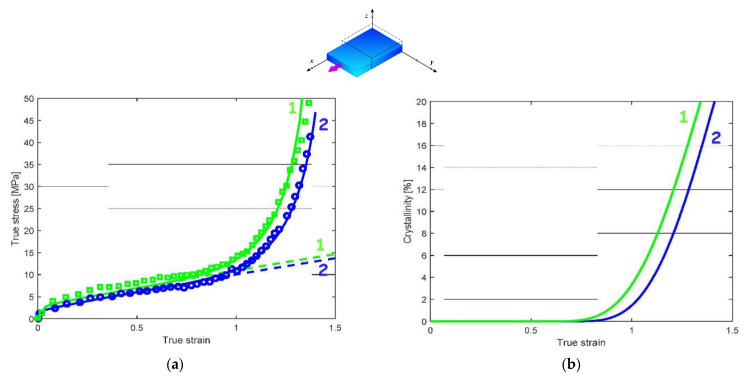
Model results under CW loading at a loading temperature of 87 °C and different strain rates (1: 1/s, 2: 4/s): (**a**) stress–strain response, (**b**) strain-induced crystallization; solid lines: model; dashed lines: model with no crystallization; symbols: experimental data of Buckley et al. [1].

Figure 4, Figure 5 and Figure 6 present the model predictions under EB and CW loadings using the model parameters identified under UA loading. The data extracted from the works of Buckley et al. [1] and Adams et al. [2] are reported in the form of symbols, while the solid lines represent the model predictions. The crystallization prediction accompanies the stress–strain curves. Note that the papers of Buckley et al. [1] and Adams et al. [2] do not present crystallization data, except at a temperature of 86 °C under EB condition.

A global view at these results shows that the model predictions are favorably compared with the experimental stress–strain curves for the two loading modes. Figure 4a and Figure 5a show that the model adequately predicts the significant effect of the straining temperature on the stress–strain response including stiffness, yield strength and strain hardening region. The predicted crystallization is also presented in Figure 4b and Figure 5b. Inversely to strain rate effects, the increase in loading temperature delays the onset of crystallization, which in turns affects the onset of strain hardening. The higher the loading temperature, the higher the strain level for which the dramatic strain hardening occurs. Figure 6 shows the theoretical and experimental CW stress–strain behavior at two strain rates: 1/s and 4/s. It can be seen that the model is able to capture the highly nonlinear mechanical response, including the dramatic strain hardening occurring at a strain of approximately 1. The higher the strain rate, the higher the onset of crystallization and the amount of crystallization.

#### 3.2.2. Implication of the Phase Changes

The phase transformation effect on the overall response can be analyzed thanks to the model considering the different loading parameters in terms of loading path, loading rate and loading temperature. When the presence of crystals is neglected in the model (dashed lines in Figure 3a, Figure 4a, Figure 5a and Figure 6a), no dramatic strain hardening occurs and the rate-dependency becomes weaker. The amorphous phase stretching may also play a role in the dramatic strain-hardening region [13]. The actual model introduces the occurrence of the crystallization as the origin of the strain hardening but, acknowledging its effective contribution [42], the supplementary effect of the amorphous phase stretching may be considered by an adequate modification of the amorphous plastic hardening in the yield function [36].

## 4. Concluding Remarks

In this work, a micromechanics-based elasto-viscoplastic constitutive model is presented. Formulated within the Eshelby inclusion theory and the micromechanics framework, the constitutive representation considers the material system as a two-phase medium in which the crystal is the reinforcement element of a viscoplastic amorphous medium which increases during stretching. The model provides a quantitative relation between deformation-induced phase transformation and mechanical response under different loading conditions in terms of loading path, loading rate and loading temperature. The model capacities are verified using available experimental observations under uniaxial, equal biaxial and constant width conditions.

Although the model can quite well capture the elasto-viscoplastic response of PET along with the strain-induced crystallization, some improvements are still necessary. It is indeed necessary to extend the mathematical description of the polymer deformation behavior to finite deformation. Furthermore, both isotropic and kinematic plastic hardening deserve to be considered in the local yield function [43] in order to bring a better description of the different steps of the overall mechanical response.

## Figures and Tables

**Table 1:** Model constants for the crystalline phase.

Parameter	Significance	Value
$E_{cry}$	Modulus	118 GPa
$\nu_{cry}$	Poisson’s ratio	0.3
α	Aspect ratio	2
${\dot{\varepsilon }}_{ref}$	Reference strain rate	2.1/s
$\phi_{\infty \_cry}$	Maximum crystal degree	0.3
$\alpha_{A}$	Avrami exponent	3
Nu	Number density of nuclei	10^8^

**Table 2:** Model constants for the amorphous phase.

Parameter	Significance	Value
$E_{g}$	Glassy modulus	2.1 GPa
$E_{r}$	Rubbery modulus	18 MPa
$\theta_{g}$	Glass transition temperature	77 °C
Δθ	Temperature shift	10 °C
$X_{g}$	Transition slope	−0.04 MPa/°C
$\nu_{g}$	Glassy Poisson’s ratio	0.32
$\nu_{r}$	Rubbery Poisson’s ratio	0.49
$\sigma_{y}$	Initial yield strength	3 MPa
h	Hardening	7 MPa
q	Hardening	0.9
η	Viscosity	0.01

## Data Availability

The data presented in this study are available on request from the corresponding author.

## Appendix A

The terms ${\bar{T}}_{1}$ and ${\bar{T}}_{2}$ of Equation (8) are expressed as:

(A1) $$\begin{array}{l} {\bar{T}}_{1}={\left(3P_{1}+2P_{2}\right)}^{2}T_{1}+2P_{1}\left(3P_{1}+4P_{2}\right)T_{2}\\ {\bar{T}}_{2}=4P_{2}^{2}T_{2} \end{array}$$

where

(A2) $$\begin{array}{l} T_{1}=\frac{1}{15}\left(T_{11}^{\left(1\right)}+4T_{12}^{\left(1\right)}+4T_{21}^{\left(1\right)}+6T_{22}^{\left(1\right)}+2T_{11}^{\left(2\right)}-4T_{12}^{\left(2\right)}+2T_{22}^{\left(2\right)}\right)\\ T_{2}=\frac{1}{15}\left(T_{11}^{\left(1\right)}-T_{12}^{\left(1\right)}-T_{21}^{\left(1\right)}+T_{22}^{\left(1\right)}+2T_{11}^{\left(2\right)}+6T_{12}^{\left(2\right)}+7T_{22}^{\left(2\right)}\right) \end{array}$$

(A3) $$\begin{array}{l} T_{IK}^{\left(1\right)}=-\frac{1}{3}+\frac{\phi^{am}}{9450{\left(1-\nu_{cry}\right)}^{2}\left(Z_{2}+S_{II}^{\left(2\right)}\right)\left(Z_{2}+S_{KK}^{\left(2\right)}\right)}[1575{\left(1-2\nu_{cry}\right)}^{2}\Gamma_{II}\Gamma_{KK}\\ \;\;\;+21\left(25\nu_{cry}-23\right)\left(1-2\nu_{cry}\right)\left(\Gamma_{II}\Delta_{K}+\Gamma_{KK}\Delta_{I}\right)+21\left(25\nu_{cry}-2\right)\\ \;\;\;\left(1-2\nu_{cry}\right)\left(\Gamma_{II}+\Gamma_{KK}\right)+3\left(35\nu_{cry}^{2}-70\nu_{cry}+36\right)\Delta_{IK}\\ \;\;\;+7\left(50\nu_{cry}^{2}-59\nu_{cry}+8\right)\left(\Delta_{I}+\Delta_{K}\right)-2\left(175\nu_{cry}^{2}-343\nu_{cry}+103\right)]\\ T_{IJ}^{\left(2\right)}=\frac{1}{2}+\frac{\phi^{am}}{6300{\left(1-\nu_{cry}\right)}^{2}\left(Z_{2}+S_{IJ}^{\left(2\right)}\right)\left(Z_{2}+S_{IJ}^{\left(2\right)}\right)}[\left(70\nu_{cry}^{2}-140\nu_{cry}-72\right)\Delta_{IJ}\\ \;\;\;-\left(175\nu_{cry}^{2}-266\nu_{cry}+75\right)\frac{\Delta_{I}+\Delta_{J}}{2}+350\nu_{cry}^{2}-476\nu_{cry}+164] \end{array}$$

(A4) $$\begin{array}{l} \Delta_{1}=\frac{3\left(1-\alpha^{4}f\left(\alpha^{2}\right)\right)}{1-\alpha^{4}},\\ \Delta_{2}=\Delta_{3}=\frac{1}{2}\left(3-\Delta_{1}\right),\\ \Delta_{11}=\frac{5\left(2+\alpha^{4}-3\alpha^{4}f\left(\alpha^{2}\right)\right)}{2{\left(1-\alpha^{4}\right)}^{2}},\\ \Delta_{12}=\Delta_{21}=\Delta_{13}=\Delta_{31}=\frac{15\alpha^{4}\left[-3+\left(1+2\alpha^{4}\right)f\left(\alpha^{2}\right)\right]}{4{\left(1-\alpha^{4}\right)}^{2}},\\ \Delta_{22}=\Delta_{23}=\Delta_{32}=\Delta_{33}=\frac{15\alpha^{4}\left[1+2\alpha^{4}+\left(1-4\alpha^{4}\right)f\left(\alpha^{2}\right)\right]}{16{\left(1-\alpha^{4}\right)}^{2}} \end{array}$$

(A5) $$f\left(\alpha \right)=\left\{\begin{array}{l} \frac{\cosh^{-1}\alpha }{\alpha \sqrt{\alpha^{2}-1}}\;\mathrm{if}\;\alpha >1\\ \frac{\cos^{-1}\alpha }{\alpha \sqrt{1-\alpha^{2}}}\;\mathrm{if}\;\alpha <1 \end{array}\right.$$

(A6) $$\begin{array}{l} P_{1}=\frac{\phi^{am}\left(\Lambda_{1}-\Omega_{1}\right)}{\left[1+2\phi^{am}\left(\Omega_{2}-\Lambda_{2}\right)\right]\left[1+\phi^{am}\left(3\Omega_{1}+2\Omega_{2}-3\Lambda_{1}-2\Lambda_{2}\right)\right]}\\ P_{2}=\frac{1}{2+4\phi^{am}\left(\Omega_{2}-\Lambda_{2}\right)} \end{array}$$

(A7) $$\begin{array}{l} \Omega_{1}=\frac{1-\Gamma_{11}-4\Gamma_{12}}{30\left(Z_{2}+S_{11}^{\left(2\right)}\right)}-\frac{1}{15\left(Z_{2}+S_{12}^{\left(2\right)}\right)}+\frac{1-4\Gamma_{21}-6\Gamma_{22}}{30\left(Z_{2}+S_{22}^{\left(2\right)}\right)}\\ \Omega_{2}=\frac{1-\Gamma_{11}+\Gamma_{12}}{30\left(Z_{2}+S_{11}^{\left(2\right)}\right)}-\frac{1}{10\left(Z_{2}+S_{12}^{\left(2\right)}\right)}+\frac{7+\Gamma_{21}-\Gamma_{22}}{60\left(Z_{2}+S_{22}^{\left(2\right)}\right)} \end{array}$$

(A8) $$\begin{array}{l} \Lambda_{1}=\frac{\left(S_{11}^{\left(1\right)}+4S_{21}^{\left(1\right)}+2S_{11}^{\left(2\right)}\right)\left(1-\Gamma_{11}-4\Gamma_{12}\right)+10S_{21}^{\left(1\right)}\Gamma_{12}}{30\left(Z_{2}+S_{11}^{\left(2\right)}\right)}-\frac{2S_{12}^{\left(2\right)}}{15\left(Z_{2}+S_{12}^{\left(2\right)}\right)}\\ \;\;+\frac{\left(3S_{22}^{\left(1\right)}+2S_{12}^{\left(1\right)}+3S_{22}^{\left(2\right)}\right)\left(3-4\Gamma_{21}-6\Gamma_{22}\right)-6S_{22}^{\left(2\right)}+5S_{12}^{\left(1\right)}\Gamma_{21}}{45\left(Z_{2}+S_{22}^{\left(2\right)}\right)}\\ \Lambda_{2}=\frac{\left(S_{11}^{\left(1\right)}-S_{21}^{\left(1\right)}+2S_{11}^{\left(2\right)}\right)\left(1-\Gamma_{11}+\Gamma_{12}\right)}{30\left(Z_{2}+S_{11}^{\left(2\right)}\right)}+\frac{S_{12}^{\left(2\right)}}{5\left(Z_{2}+S_{12}^{\left(2\right)}\right)}\\ \;\;+\frac{\left(S_{22}^{\left(1\right)}-S_{12}^{\left(1\right)}+S_{22}^{\left(2\right)}\right)\left(1+2\Gamma_{21}-2\Gamma_{22}\right)+6S_{22}^{\left(1\right)}}{30\left(Z_{2}+S_{22}^{\left(2\right)}\right)} \end{array}$$

(A9) $$\begin{array}{l} \Gamma_{I1}=\frac{\left(Z_{1}+Z_{2}+S_{22}^{\left(1\right)}+S_{22}^{\left(2\right)}\right)\left(Z_{1}+S_{I1}^{\left(1\right)}\right)-\left(Z_{1}+S_{21}^{\left(1\right)}\right)\left(Z_{1}+S_{I2}^{\left(1\right)}\right)}{\left(Z_{1}+Z_{2}+S_{22}^{\left(1\right)}+S_{22}^{\left(2\right)}\right)\left(Z_{1}+2Z_{2}+S_{11}^{\left(1\right)}+2S_{11}^{\left(2\right)}\right)-\left(Z_{1}+S_{12}^{\left(1\right)}\right)\left(Z_{1}+S_{21}^{\left(1\right)}\right)}\\ \Gamma_{I2}=\Gamma_{I3}=\frac{\left(Z_{1}+2Z_{2}+S_{11}^{\left(1\right)}+2S_{11}^{\left(2\right)}\right)\left(Z_{1}+S_{I2}^{\left(1\right)}\right)-\left(Z_{1}+S_{12}^{\left(1\right)}\right)\left(Z_{1}+S_{I1}^{\left(1\right)}\right)}{2\left(Z_{1}+Z_{2}+S_{22}^{\left(1\right)}+S_{22}^{\left(2\right)}\right)\left(Z_{1}+2Z_{2}+S_{11}^{\left(1\right)}+2S_{11}^{\left(2\right)}\right)-2\left(Z_{1}+S_{12}^{\left(1\right)}\right)\left(Z_{1}+S_{21}^{\left(1\right)}\right)} \end{array}$$

(A10) $$\begin{array}{l} Z_{1}=\frac{\lambda_{cry}\mu_{am}-\lambda_{am}\mu_{cry}}{\left(\mu_{am}-\mu_{cry}\right)\left[2\left(\mu_{am}-\mu_{cry}\right)+3\left(\lambda_{am}-\lambda_{cry}\right)\right]}\\ Z_{2}=\frac{\mu_{cry}}{2\left(\mu_{am}-\mu_{cry}\right)} \end{array}$$

(A11) $$\begin{array}{l} \lambda_{cry}=\frac{E_{cry}\nu_{cry}}{\left(1+\nu_{cry}\right)\left(1-2\nu_{cry}\right)},\;\;\mu_{cry}=\frac{E_{cry}}{2\left(1+\nu_{cry}\right)}\\ \lambda_{am}=\frac{E_{am}\nu_{am}}{\left(1+\nu_{am}\right)\left(1-2\nu_{am}\right)},\;\;\mu_{am}=\frac{E_{am}}{2\left(1+\nu_{am}\right)} \end{array}$$

(A12) $$\begin{array}{l} S_{11}^{\left(1\right)}=\left(4\nu_{cry}+\frac{2}{\alpha^{2}-1}\right)g\left(\alpha \right)+4\nu_{cry}+\frac{4}{3\left(\alpha^{2}-1\right)}\\ S_{12}^{\left(1\right)}=S_{13}^{\left(1\right)}=\left(4\nu_{cry}+\frac{2\alpha^{2}+1}{\alpha^{2}-1}\right)g\left(\alpha \right)+4\nu_{cry}-\frac{2\alpha^{2}}{\alpha^{2}-1}\\ S_{21}^{\left(1\right)}=S_{31}^{\left(1\right)}=\left(-2\nu_{cry}-\frac{2\alpha^{2}+1}{\alpha^{2}-1}\right)g\left(\alpha \right)-\frac{2\alpha^{2}}{\alpha^{2}-1}\\ S_{22}^{\left(1\right)}=S_{23}^{\left(1\right)}=S_{32}^{\left(1\right)}=S_{33}^{\left(1\right)}=\left(-2\nu_{cry}+\frac{4\alpha^{2}-1}{4\left(\alpha^{2}-1\right)}\right)g\left(\alpha \right)+\frac{\alpha^{2}}{2\left(\alpha^{2}-1\right)}\\ S_{11}^{\left(2\right)}=\left(-4\nu_{cry}+\frac{4\alpha^{2}-2}{\alpha^{2}-1}\right)g\left(\alpha \right)-4\nu_{cry}+\frac{12\alpha^{2}-8}{3\left(\alpha^{2}-1\right)}\\ S_{12}^{\left(2\right)}=S_{13}^{\left(2\right)}=S_{21}^{\left(2\right)}=S_{31}^{\left(2\right)}=\left(-\nu_{cry}-\frac{\alpha^{2}+2}{\alpha^{2}-1}\right)g\left(\alpha \right)-2\nu_{cry}-\frac{2}{\alpha^{2}-1}\\ S_{22}^{\left(2\right)}=S_{23}^{\left(2\right)}=S_{32}^{\left(2\right)}=S_{33}^{\left(2\right)}=\left(2\nu_{cry}-\frac{4\alpha^{2}-7}{4\left(\alpha^{2}-1\right)}\right)g\left(\alpha \right)+\frac{\alpha^{2}}{2\left(\alpha^{2}-1\right)} \end{array}$$

(A13) $$g\left(\alpha \right)=\left\{\begin{array}{l} \frac{\alpha }{{\left(\alpha^{2}-1\right)}^{3/2}}\left[\cosh^{-1}\alpha -\alpha {\left(\alpha^{2}-1\right)}^{1/2}\right]\;\mathrm{if}\;\alpha >1\\ \frac{\alpha }{{\left(1-\alpha^{2}\right)}^{3/2}}\left[\alpha {\left(1-\alpha^{2}\right)}^{1/2}-\cos^{-1}\alpha \right]\;\mathrm{if}\;\alpha <1 \end{array}\right.$$

## References

[1] Buckley C.P., Jones D.C., Jones D.P. (1996). Hot-drawing of poly(ethylene terephthalate) under biaxial stress: Application of a three-dimensional glass-rubber constitutive model. Polymer.

[2] Adams A.M., Buckley C.P., Jones D.P. (2000). Biaxial hot drawing of poly(ethylene terephthalate): Measurements and modelling of strain-stiffening. Polymer.

[3] Boyce M.C., Socrate S., Llana P.G. (2000). Constitutive model for the finite deformation stress-strain behavior of poly(ethylene terephthalate) above the glass transition. Polymer.

[4] Doufas A.K., McHugh A.J., Miller C. (2000). Simulation of melt spinning including flow-induced crystallization: Part I. Model development and predictions. J. Non-Newton. Fluid Mech..

[5] Ahzi S., Makradi A., Gregory R.V., Edie D.D. (2003). Modeling of deformation behavior and strain-induced crystallization in poly(ethylene terephthalate) above the glass transition temperature. Mech. Mater..

[6] Makradi A., Ahzi S., Gregory R.V., Edie D.D. (2005). A two-phase self-consistent model for the deformation and phase transformation behavior of polymers above the glass transition temperature: Application to PET. Int. J. Plast..

[7] Dupaix R.B., Krishnan D. (2006). A constitutive model for strain-induced crystallization in poly(ethylene terephthalate) (PET) during finite strain load-hold simulations. J. Eng. Mater. Technol..

[8] Dupaix R.B., Boyce M.C. (2007). Constitutive modeling of the finite strain behavior of amorphous polymers in and above the glass transition. Mech. Mater..

[9] Figiel L., Buckley C.P. (2009). On the modelling of highly elastic flows of amorphous thermoplastics. Int. J. Non-Linear Mech..

[10] Chevalier L., Luo Y.M., Monteiro E., Menary G.H. (2012). On visco-elastic modelling of polyethylene terephthalate behaviour during multiaxial elongations slightly over the glass transition temperature. Mech. Mater..

[11] Cosson B., Chevalier L., Régnier G. (2012). Simulation of the stretch blow moulding process: From the modelling of the microstructure evolution to the end-use elastic properties of polyethylene terephthalate bottles. Int. J. Mater. Form..

[12] Menary G.H., Tan C.W., Harkin-Jones E.M.A., Armstrong C.G., Martin P.J. (2012). Biaxial deformation and experimental study of PET at conditions applicable to stretch blow molding. Polym. Eng. Sci..

[13] Mahjoubi H., Zaïri F., Tourki Z. (2019). A micro-macro constitutive model for strain-induced molecular ordering in biopolymers: Application to polylactide over a wide range of temperatures. Int. J. Plast..

[14] Mahjoubi H., Zaïri F., Tourki Z. (2020). Strain-induced phase transformation in poly(lacticacid) across the glass transition: Constitutive model and identification. Int. J. Non-Linear Mech..

[15] Ayoub G., Zaïri F., Naït-Abdelaziz M., Gloaguen J.M. (2010). Modelling large deformation behaviour under loading-unloading of semicrystalline polymers: Application to a high density polyethylene. Int. J. Plast..

[16] Ayoub G., Zaïri F., Fréderix C., Gloaguen J.M., Naït-Abdelaziz M., Seguela R., Lefebvre J.M. (2011). Effects of crystal content on the mechanical behaviour of polyethylene under finite strains: Experiments and constitutive modelling. Int. J. Plast..

[17] Abdul-Hameed H., Messager T., Zaïri F., Naït-Abdelaziz M. (2014). Large-strain viscoelastic-viscoplastic constitutive modeling of semi-crystalline polymers and model identification by deterministic/evolutionary approach. Comput. Mater. Sci..

[18] Makki M., Ayoub G., Abdul-Hameed H., Zaïri F., Mansoor B., Naït-Abdelaziz M., Ouederni M., Zaïri F. (2017). Mullins effect in polyethylene and its dependency on crystal content: A network alteration model. J. Mech. Behav. Biomed. Mater..

[19] Bernard C.A., Lame O., Deplancke T., Cavaillé J.Y., Ogawa K. (2020). From rheological to original three-dimensional mechanical modelling of semi-crystalline polymers: Application to a wide strain rate range and large deformation of Ultra-High Molecular Weight PolyEthylene. Mech. Mater..

[20] Lee B.J., Parks D.M., Ahzi S. (1993). Micromechanical modeling of large plastic deformation and texture evolution in semi-crystalline polymers. J. Mech. Phys. Solids.

[21] Lee B.J., Argon A.S., Parks D.M., Ahzi S., Bartczak Z. (1993). Simulation of large strain plastic deformation and texture evolution in high density polyethylene. Polymer.

[22] Nikolov S., Doghri I., Pierard O., Zealouk L., Goldberg A. (2002). Multi-scale constitutive modeling of the small deformations of semi-crystalline polymers. J. Mech. Phys. Solids.

[23] Van Dommelen J.A.W., Parks D.M., Boyce M.C., Brekelmans W.A.M., Baaijens F.P.T. (2003). Micromechanical modeling of the elasto-viscoplastic behavior of semi-crystalline polymers. J. Mech. Phys. Solids.

[24] Agoras M., Ponte Castaneda P. (2012). Multi-scale homogenization-based modeling of semi-crystalline polymers. Philos. Mag..

[25] Poluektov M., van Dommelen J.A.W., Govaert L.E., MacKerron D.H., Geers M.G.D. (2016). Micromechanical modeling of roll-to-roll processing of oriented polyethylene terephthalate films. J. Appl. Polym. Sci..

[26] Bedoui F., Diani J., Régnier G. (2004). Micromechanical modeling of elastic properties in polyolefins. Polymer.

[27] Sedighiamiri A., Van Erp T.B., Peters G.W.M., Govaert L.E., van Dommelen J.A.W. (2010). Micromechanical modeling of the elastic properties of semicrystalline polymers: A three-phase approach. J. Polym. Sci. Part B Polym. Phys..

[28] Bedoui F., Diani J., Régnier G., Seiler W. (2006). Micromechanical modeling of isotropic elastic behavior of semicrystalline polymers. Acta Mater..

[29] Guan X., Pitchumani R. (2004). A micromechanical model for the elastic properties of semicrystalline thermoplastic polymers. Polym. Eng. Sci..

[30] Ahzi S., Bahlouli N., Makradi A., Belouettar S. (2007). Composite modeling for the effective elastic properties of semicrystalline polymers. J. Mech. Mater. Struct..

[31] Gueguen O., Ahzi S., Makradi A., Belouettar S. (2010). A new three-phase model to estimate the effective elastic properties of semi-crystalline polymers: Application to PET. Mech. Mater..

[32] Anoukou K., Zaïri F., Naït-Abdelaziz M., Zaoui A., Qu Z., Gloaguen J.M., Lefebvre J.M. (2014). A micromechanical model taking into account the contribution of α- and γ-crystalline phases in the stiffening of polyamide 6-clay nanocomposites: A closed-formulation including the crystal symmetry. Compos. Part B Eng..

[33] Yao S., Hu D., Xi Z., Liu T., Xu Z., Zhao L. (2020). Effect of crystallization on tensile mechanical properties of PET foam: Experiment and model prediction. Polym. Test..

[34] Bedoui F., Guigon M. (2010). Linear viscoelastic behavior of poly(ethylene terephtalate) above T_g_ amorphous viscoelastic properties Vs crystallinity: Experimental and micromechanical modeling. Polymer.

[35] Hachour K., Zaïri F., Naït-Abdelaziz M., Gloaguen J.M., Aberkane M., Lefebvre J.M. (2014). Experiments and modeling of high-crystalline polyethylene yielding under different stress states. Int. J. Plast..

[36] Mesbah A., Elmeguenni M., Yan Z., Zaïri F., Ding N., Gloaguen J.M. (2021). How stress triaxiality affects cavitation damage in high-density polyethylene: Experiments and constitutive modeling. Polym. Test..

[37] Liu H.T., Sun L.Z. (2005). Multi-scale modeling of elastoplastic deformation and strengthening mechanisms in aluminium-based amorphous nanocomposites. Acta Mater..

[38] Ju J.W., Sun L.Z. (2001). Effective elastoplastic behavior of metal matrix composites containing randomly located aligned spheroidal inhomogeneities. Part I: Micromechanics-based formulation. Int. J. Solids Struct..

[39] Simo J.C., Kennedy J.G., Govindjee S. (1988). Non-smooth multisurface plasticity and viscoplasticity. Loading/unloading conditions and numerical algorithms. Int. J. Numer. Methods Eng..

[40] Ju J.W., Zhang X.D. (2001). Effective elastoplastic behavior of ductile matrix composites containing randomly located aligned circular fibers. Int. J. Solids Struct..

[41] Salem D.R. (1992). Development of crystalline order during hot-drawing of poly(ethylene terephthalate) film: Influence of strain rate. Polymer.

[42] Yan Z., Guo Q., Zaïri F., Zaoui A., Jiang Q., Liu X. (2021). Continuum-based modeling large-strain plastic deformation of semi-crystalline polyethylene systems: Implication of texturing and amorphicity. Mech. Mater..

[43] Basiri A., Zaïri F., Azadi M., Ghasemi-Ghalebahman A. (2022). Micromechanical constitutive modeling of tensile and cyclic behaviors of nano-clay reinforced metal matrix nanocomposites. Mech. Mater..

